# Mendelian randomization study and meta‐analysis exploring the causality of age at menarche and the risk of intracerebral hemorrhage and ischemic stroke

**DOI:** 10.1111/cns.14245

**Published:** 2023-05-11

**Authors:** Xuelun Zou, Leiyun Wang, Sai Wang, Le Zhang

**Affiliations:** ^1^ Department of Neurology Xiangya Hospital, Central South University Changsha Hunan China; ^2^ Department of Pharmacy Wuhan First Hospital Wuhan China; ^3^ National Clinical Research Center for Geriatric Disorders, Xiangya Hospital Central South University Changsha Hunan China; ^4^ Multi‐Modal Monitoring Technology for Severe Cerebrovascular Disease of Human Engineering Research Center, Xiangya Hospital Central South University Changsha Hunan China

**Keywords:** age at menarche, genome‐wide association analysis, intracerebral hemorrhage, ischemic stroke, mendelian randomization, meta‐analysis

## Abstract

**Background:**

The relationship between the age at menarche (AAM) and the risk of intracerebral hemorrhage (ICH) and ischemic stroke (IS) is still up for debate. The purpose of this study was to investigate potential causal connections between them.

**Methods:**

Genome‐wide association analysis (GWAS) of AAM conducted by the MRC‐IEU consortium was utilized for association analyses of ICH and IS by two‐sample Mendelian randomization (MR) study. AAM data of the within‐family GWAS consortium were used as replication phase data to verify the causal relationship between each other. Inverse variance weighting (IVW) method was the primary method used in this MR study. For additional proof, the weighted median estimation, MR‐Egger regression, MR‐PRESSO test, and MR‐Robust Adjusted Profile Score evaluation were performed. The Cochran's *Q* test and the MR‐PRESSO global test were used, respectively, to examine the sensitivity and pleiotropy. Random effects meta‐analysis was utilized to analyze the causal data from the two consortiums to further explore the causality between AAM and ICH, IS.

**Results:**

We found that the AAM was causally linked with the risk of ICH (OR = 0.48, 95% CI: 0.28–0.80, *p* = 0.006). On the contrary, the causal effect from AAM to IS (OR = 0.98, 95% CI: 0.91–1.06, *p* = 0.64) has not been confirmed. For all subtypes of ICH, we found that nonlobar intracerebral hemorrhage (NLICH, OR = 0.41, 95% CI: 0.23–0.75, *p* = 0.004) but not lobar intracerebral hemorrhage (LICH, OR = 0.65, 95% CI: 0.34–1.24, *p* = 0.19) was associated with AAM without surprise. Similarly, we used the within‐family GWAS consortium data to explore causality and found that AAM may reduce the risk of ICH (OR = 0.78, 95% CI: 0.72–0.86, *p* = 9.5 × 10^−8^) and NLICH (OR = 0.68, 95% CI: 0.61–0.75, *p* = 3.4 × 10^−13^) by IVW methods, but is not related to IS (OR = 0.97, 95% CI: 0.93–1.02, *p* = 0.26). These findings are further supported by the meta‐analysis. Both Cochran's *Q* test and the MR‐PRESSO global test failed to detect the presence of sensitivity.

**Conclusion:**

AAM and ICH, particularly NLICH, are causally related, but not LICH, IS, or its subtypes in European population.

## Graphical abstract

Research design of this study. ICH, intracerebral hemorrhage; LICH, lobar intracerebral hemorrhage; NLICH, nonlobar intracerebral hemorrhage; IS, ischemic stroke; LAS, large artery stroke; CES, cardioembolic stroke; SVS, small vessel stroke; WFG, within‐family GWAS; ISG, International Stroke Genetics.

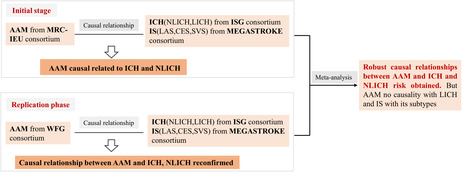

## 1 INTRODUCTION

Age at menarche (AAM) refers to the age at which puberty women first exhibit endometrial bleeding brought on by the regulation of estrogen and progesterone, which signals the start of women's menstrual cycle. More and more research has shown that women's AAM is gradually earlier as a result of lifestyle changes.^1^, ^2^ Critical illnesses like obesity,^1^ type II diabetes,^2^ cardiovascular disease,^3^, ^4^ and cancers of the reproductive system like breast cancer and ovarian cancer^5^, ^6^ in the female population have also been linked to early AAM. Premature AAM is closely linked to the effects of estrogen and progesterone and may be influenced by them as a potential cause of many female diseases.

Estrogen and progesterone may have important effects on stroke, such as female pregnancy,^7^, ^8^ birth rate,^8^, ^9^ menopause,^9^ and external use of these two hormones.^9^ The second largest cause of death and disability worldwide is stroke, which is also the third leading cause of death and disability.^10^ Stroke poses a severe hazard to people's health all over the world with a prevalence of 101 million and a death toll of 6.55 million.^10^ Intracerebral hemorrhage (ICH) and ischemic stroke (IS) are the two main subtypes of stroke but differ markedly in their pathogenesis (vascular rupture/vascular embolism). There are also significant gender differences in the incidence and mortality rates of the two diseases, and the effects of estrogen and progesterone may be important in accounting for this difference.^9^, ^11^, ^12^

Age at menarche is the time when the body is first affected by estrogen and progesterone, and it affects the length of a woman's exposure to estrogen and progesterone and her reproductive life span. It is the earliest indication of the effects of hormones compared with pregnancy and menopause and provides the best predictor of future prevention and control. Therefore, exploring the interrelationship between AAM and ICH/IS has significant clinical indications.

A multicenter case–control study showed that women with first menstruation younger than 13 years old had a higher risk of developing IS (OR = 1.64, 95% CI: 1.11–2.17).^13^ Conversely, a meta‐analysis of 75 studies found that women with shorter reproductive cycles had a higher risk of stroke (OR = 1.31, 95% CI: 1.25–1.36), which indicates that the earlier the AAM, the lower the risk of stroke.^14^ Furthermore, according to a cohort study of 1.2 million adults in the UK, the association between AAM and stroke was *U*‐shaped, with the risk of stroke rising with age between ≥17 and ≤10.^15^ Taken together, the relationship between AAM and IS risk is controversial (IS accounts for more than 80% of strokes). Also, the relationship between AAM and the risk of ICH is unclear.

Mendelian randomization (MR) is frequently used to assess the effects of confounding factors in epidemiological etiology on inferences.^16^ MR can be used to directly analyze the causal relationship between exposure and outcome through genetic variables, eradicating the influence of confounding factors and reversing causality on the outcomes with the aid of GWAS data.^17^ On the above background, we want to use the novel method of MR to explore the causal relationship between AAM and the risk of IS and ICH from a genetic perspective, and meta‐analysis was used to integrate the results of the two different consortiums to obtain a robust causal relationship.

## 2 METHODS

### 2.1 Study design

**FIGURE 1:**
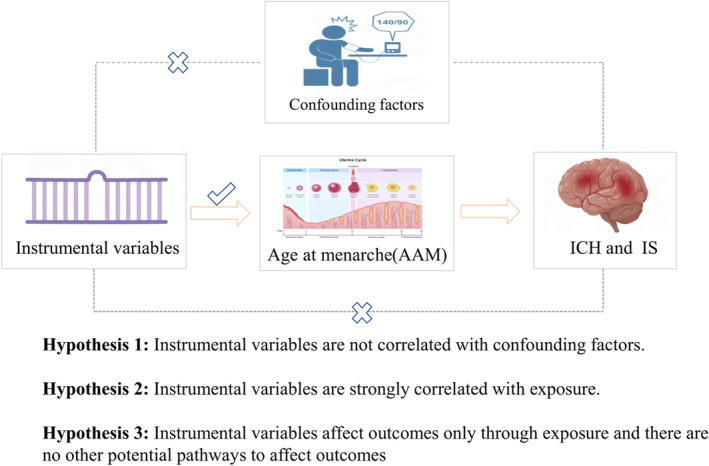
Three core assumptions of AAM with ICH and IS in this two‐sample MR study. AAM, age at menarche; CES, cardioembolic stroke; ICH, intracerebral hemorrhage; IS, ischemic stroke; LAS, large artery stroke; LICH, lobar intracerebral hemorrhage; NLICH, nonlobar intracerebral hemorrhage; SVS, small vessel stroke.

**FIGURE 2:**
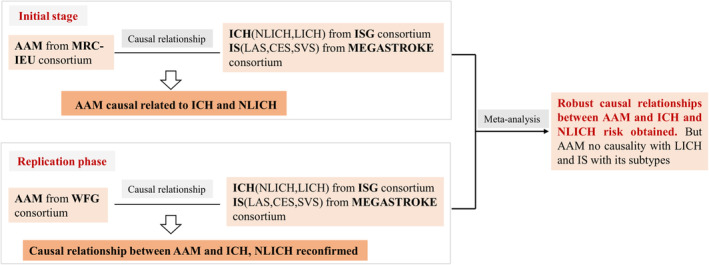
Research design of this study. In the initial stage, the MRC‐IEU Consortium was used to obtain GWAS data of AAM as exposure data, and the MEGASTROKE consortium obtained GWAS data of IS and ICH as outcome data to analyze the causal relationship between AAM and IS, ICH, and their subtypes. In the replication phase, the WFG Consortium was used to obtain GWAS data for AAM as exposure data, and the MEGASTROKE consortium obtained GWAS data for IS and ICH as outcome data to analyze the causal relationship between AAM and IS, ICH, and their subtypes. Finally, the causal data obtained by MRC‐IEU and WFG consortium were meta‐analyzed to obtain more robust causal relationships. CES, cardioembolic stroke; ICH, intracerebral hemorrhage; IS, ischemic stroke; ISG, International Stroke Genetics; LAS, large artery stroke; LICH, lobar intracerebral hemorrhage; NLICH, nonlobar intracerebral hemorrhage; SVS, small vessel stroke; WFG, within‐family GWAS.

Aiming to elucidate the relationship between AAM and the stroke subtypes of IS and ICH, we designed this main MR investigation. Additionally, several sensitivity analysis techniques were performed to guarantee the reliability of the findings (Figure 1). Data from another GWAS database were then used to validate the causal relationship between AAM and the risk of ICH and IS. Finally, a meta‐analysis was conducted to integrate the causal relationships explored by different GWAS data between AAM and ICH, IS, and their subtypes to obtain a robust causal relationship (Figure 2). The public database was used to obtain all the study's data.^18^ All the study subjects provided their informed consent, and the design of the investigations passed an ethical assessment. Therefore, further ethical evaluation of this work is not necessary.

### 2.2 Sources of exposure and outcome

The GWAS database run by the MRC‐IEU consortium served as the initial source of exposure data (AAM) for this investigation in 2018.^19^ There were 243,944 individuals overall in this study who were of European descent. The validated data came from a large cohort study of 159,701 siblings by the within‐family GWAS consortium that was published in 2022. Of these samples, 29,346 underwent GWAS at the AAM and 7,890,254 single‐nucleotide loci were analyzed (Table 1).

**TABLE 1:** Data for including exposure and outcome information in GWAS.

Phenotype	Number of SNP	Cases	Controls	Sample size	Population	Consortium
Exposure						
AAM (Initial stage)	9,851,867	NA	NA	243,944	European	MRC‐IEU
AAM (Replication phase)	7,890,254	NA	NA	29,346	European	Within‐family GWAS
Outcome						
ICH	NA	1545	1481	3026	European	International Stroke Genetics
LICH	NA	686	1481	2167	European	International Stroke Genetics
NLICH	NA	909	1481	2390	European	International Stroke Genetics
IS	7,537,579	34,217	406,111	440,328	European	MEGASTROKE
SVS	6,150,261	5386	192,662	198,048	European	MEGASTROKE
CES	7,954,834	7193	406,111	211,763	European	MEGASTROKE
LAS	7,992,739	4373	406,111	150,765	European	MEGASTROKE

Abbreviations: CES, cardioembolic stroke; CH, intracerebral hemorrhage; IS, ischemic stroke; LAS, large artery atherosclerosis stroke; LICH, lobar intracerebral hemorrhage; NLICH, nonlobar intracerebral hemorrhage; SVS, small vessel stroke.

The outcome (IS and ICH) data in this study was extracted from the MEGASTROKE consortium's 2018 meta‐analysis of 29 strokes GWAS data,^20^ which includes 40, 585 patients and 406, 111 controls. There were 34,217 cases of the IS subtype, as opposed to 4373 cases each of large artery stroke (LAS), 7913 cases each of cardioembolic stroke (CES), 5386 cases each of small vessel stroke (SVS), and some unidentified stroke included in the database. The International Stroke Genetics Consortium's three GWAS of ICH, which comprised 1, 545 cases and 1, 481 European‐born healthy controls, provided the ICH data.^20^ These cases were grouped into two categories of ICH: lobar intracerebral hemorrhage (LICH, 686 instances) and nonlobar intracerebral hemorrhage (NLICH, 909 cases).

### 2.3 Two‐Sample Mendelian randomization

Instrumental variables, namely single‐nucleotide polymorphisms (SNPs), are extracted from GWAS data of exposure variables. The significant effect of extracting instrumental variables is defined as the *p*‐value of SNP less than 5 × 10^−8^. On the contrary, it cannot be ignored that the existence of linkage disequilibrium will affect the analysis quality. Therefore, we defined the criteria for removing linkage disequilibrium as *r* ^2^ < 0.01 and kb as 10,000. All included SNPs and excluded SNPs are shown in Tables S1 and S2.

Currently, the IVW approach has been chosen as the primary analysis method in this research.^21^ This technique can be used to determine whether exposure and outcome have a consistent and reliable causal relationship. But for it to produce consistent findings, all genetic variations must be treated as instrumental variables. In addition, a few more techniques are utilized to support the IVW method and offer more proof for this study's causality assessment. Even with 50% invalid instrumental variables, the weighted median estimate (WME) approach can still produce trustworthy findings.^22^ Although directional pleiotropic bias can be controlled via MR‐Egger regression, its assessment effect is not very strong.^23^ A new evaluation technique called MR‐Pleiotropy Residual Sum and Outlier (MR‐PRESSO), created in 2018, can identify and correct outliers in each instrumental variable.^24^ It can also analyze causation and find directional pleiotropy. A reliable method that can remove the effects of weak instrumental factors, systemic pleiotropy, and idiosyncratic pleiotropy on causality, known as the MR‐Robust Adjusted Profile Score (MR.RAPS)^25^ has been adopted in this study.

A series of sensitivity analyses are employed to determine whether the causality assessed by the above methods has heterogeneity, directional pleiotropy, etc. To ascertain whether each instrumental variable is heterogeneous or not, the heterogeneity test (also known as Cochran's *Q* test) is used. To mitigate the effect of heterogeneity on the results, we must use a random effect model. The *p*‐value of the global test in MR‐PRESSO and the intercept MR‐Egger regression was used to determine the directionality of pleiotropy.^23^, ^24^ Both the MR‐Egger regression approach and the IVW method did not perform the actual evaluation, known as the no measurement error (NOME) assessment, but instead simply took into account the association between SNPs and exposure. Therefore, it must be >90% for I2GX to evaluate the NOME of MR‐Egger regression.^26^ F‐statistics, a tool to identify the presence of weak instrumental factors and assess the efficacy of research, is used to estimate the NOME in the IVW approach.^27^ Table S1 is used to display these findings.

### 2.4 Meta‐analysis

Finally, we performed a random effects meta‐analysis of the results (OR, 95% UL, 95% LL) obtained from the MRC‐IEU and within‐family GWAS data to obtain a combined analysis of causality. The meta‐analysis was carried out in STATA software version 14.0, and the statistical criterion for meta‐analysis results was set at 0.05; similarly, the statistical criterion for the test of heterogeneity was also 0.05.

### 2.5 Statistical analysis

All the statistical analyses in the MR study were conducted using R studio and R 4.0.3, including the “Mendelian randomization” package and the “TwoSampleMR” package. AAM and stroke subtypes' statistical significance was assessed using the stringent statistical approach of Bonferroni correction.^28^ AAM and ICH and its two ICH subtypes had a *p* < 0.016 (0.05/3), and AAM and IS and all of its four subtypes had a *p* < 0.0125 (0.05/4).

## 3 RESULTS

### 3.1 The AAM was related to the risk of ICH, especially NLICH and was validated by the results of the within‐family GWAS consortium data and meta‐analysis

**FIGURE 3:**
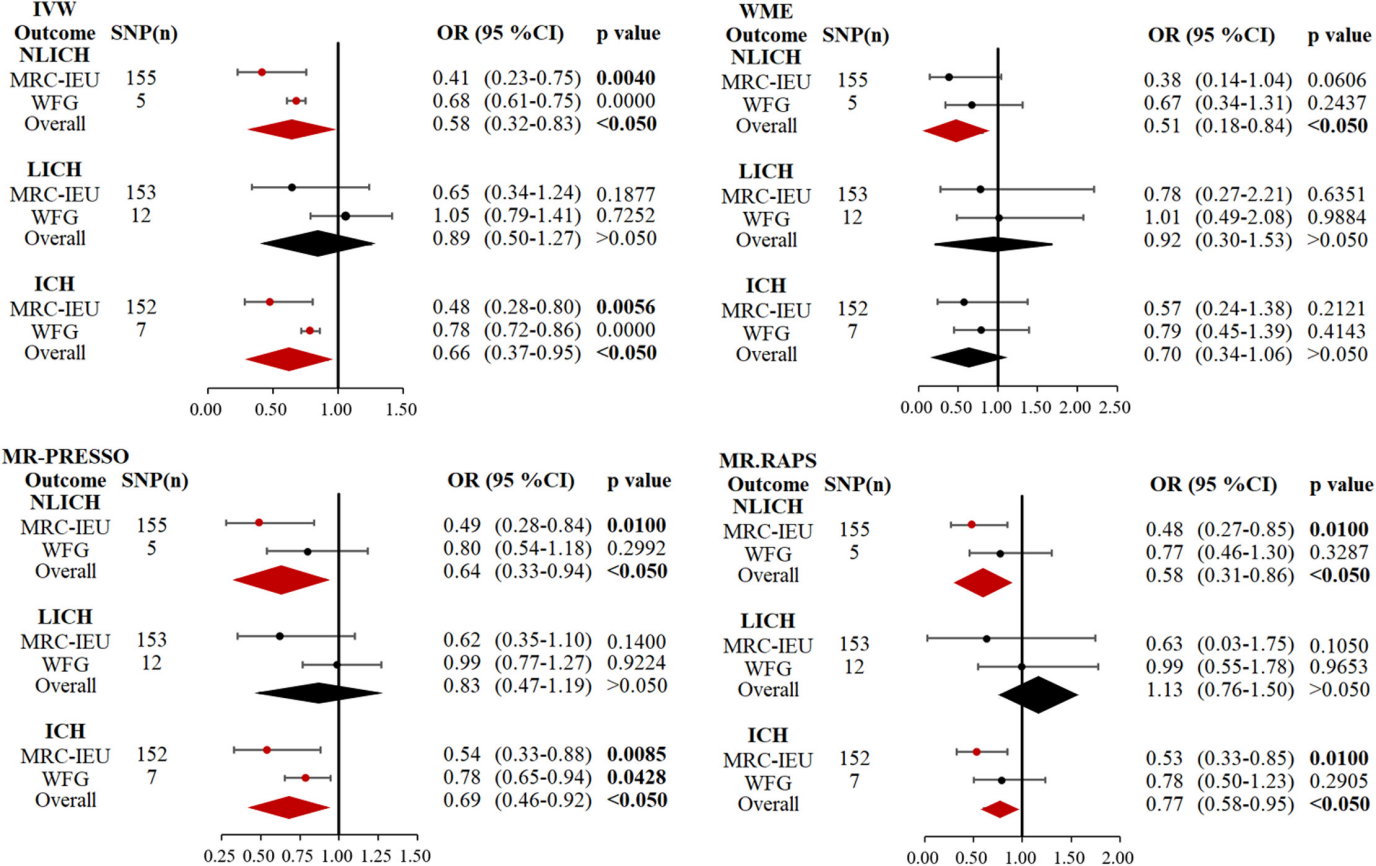
Causal relationships between AAM and ICH with its subtypes by IVW, WME, MR.RAPS, MR‐PRESSO methods in the initial phase, replication phase, and meta‐analysis. A causal relationship between AAM and NLICH was found in the IVW, WME, MR‐PRESSO, and MR.RAPS methods after the meta‐analysis of causality in the initial and replication phases. In addition, the causal relationship between AAM and ICH was also confirmed in the meta‐analysis of the IVW, MR‐PRESSO, and MR.RAPS methods. AAM, age at menarche; ICH, intracerebral hemorrhage; IVW, inverse‐variance‐weighted; NLICH, nonlobar intracerebral hemorrhage; SNP(*n*), SNP number; WFG, within‐family GWAS; WME, weighted median estimation.

In the initial phase of the MR analysis employing MRC‐IEU data, AAM and the risk of ICH (OR = 0.48, 95% CI: 0.28–0.80, *p* = 0.006) were strongly associated, as shown in Table 2 and Figure 3, in the IVW fixed‐effect model. The MR‐PRESSO (OR = 0.53, 95% CI: 0.33–0.85, *p* = 0.009) and MR.RAPS (OR = 0.54, 95% CI: 0.33–0.88, *p* = 0.01) methods similarly demonstrated causal evidence of AAM and the risk of ICH. The MR study did demonstrate a causal relationship between AAM and the risk of NLICH (OR = 0.41, 95% CI: 0.23–0.75, *p* = 0.004, Figure 3) in the ICH subtypes. Furthermore, using the MR‐PRESSO (OR = 0.49, 95% CI: 0.28–0.84, *p* = 0.01) and MR.RAPS (OR = 0.48, 95% CI: 0.27–0.85, *p* = 0.01) methods, the causal relationship between AAM and the risk of NLICH was also discovered. However, there was no connection between AAM and the risk of LICH (OR = 0.65, 95% CI: 0.34–1.24, *p* = 0.19) in the fixed‐effect IVW approach.

**TABLE 2:** Causality of AAM with ICH and IS with its subtypes by IVW method (MRC‐IEU).

	AAM (MRC‐IEU)			
	SNP	OR	95% CI (LL–UL)	*p*‐value
ICH	152	**0.53**	**0.33–0.85**	**0.0100**
NLICH	155	**0.41**	**0.23–0.75**	**0.0040**
LICH	153	0.63	0.03–1.75	0.1050
IS	254	0.98	0.91–1.06	0.6638
SVS	229	0.95	0.81–1.11	0.5013
CES	258	1.00	0.85–1.16	0.9579
LAS	257	0.89	0.75–1.07	0.2251

*Note*: In the initial phase, a causal relationship between AAM and ICH and NLICH was identified. The bold values represent statistically significant causal relationships in the initial phase.

Abbreviations: AAM, age at menarche; CES, cardioembolic stroke; IS, ischemic stroke; ICH, intracerebral hemorrhage; LAS, large artery atherosclerosis stroke; LICH, lobar intracerebral hemorrhage; NLICH, nonlobar intracerebral hemorrhage; SNP, single‐nucleotide polymorphism; SVS, small vessel stroke.

Moreover, the within‐family GWAS consortium of the replication phase reported that AMM was associated with ICH (OR = 0.78, 95% CI: 0.72–0.86, *p* = 9.5 × 10^−8^) and NLICH (OR = 0.68, 95% CI: 0.61–0.75, *p* = 3.4 × 10^−13^) but not LICH (OR = 1.05, 95% CI: 0.79–1.41, *p* = 0.73; Table 3 and Figure 2). AAM may be related to the risk of NLICH, according to the results of the IVW (OR = 0.58, 95% CI: 0.32–0.83, *p* < 0.05), WME (OR = 0.51, 95% CI: 0.18–0.84, *p* < 0.05), MR‐PRESSO (OR = 0.64, 95% CI: 0.33–0.94, *p* < 0.05), and MR.RAPS (OR = 0.58, 95% CI: 0.31–0.86, *p* < 0.05). Additionally, the IVW (OR = 0.66, 95% CI: 0.37–0.95, *p* < 0.05), MR‐PRESSO (OR = 0.69, 95% CI: 0.46–0.92, *p* < 0.05), and MR.RAPS (OR = 0.77, 95% CI: 0.58–0.95, *p* < 0.05) studies investigated a causative association between AAM and the risk of ICH. In the meta‐analysis, the causal link between AAM and the risk of ICH and NLICH was also established (as shown in Figure 3). These support the reliability of the findings from our MR study.

**TABLE 3:** Causality of AAM with ICH and IS with its subtypes by IVW method (within‐family GWAS consortium).

	AAM (MRC‐IEU)			
	SNP	OR	95% CI (LL–UL)	*p*‐value
ICH	5	**0.53**	**0.33–0.85**	**3.40 × 10** ^**−13**^
NLICH	5	**0.78**	**0.72–0.86**	**9.51 × 10** ^**−8**^
LICH	5	1.05	0.79–1.41	0.7252
IS	5	0.97	0.92–1.02	0.2545
SVS	5	1.00	0.80–1.24	0.9853
CES	5	1.01	0.79–1.30	0.9095
LAS	5	0.97	0.83–1.13	0.6960

*Note*: During the replication phase, a causal relationship between AAM and ICH, NLICH was identified. The bold values represent statistically significant causal relationships in the replication phase.

Abbreviations: AAM, age at menarche; CES, cardioembolic stroke; ICH, intracerebral hemorrhage; IS, ischemic stroke; LAS, large artery atherosclerosis stroke; LICH, lobar intracerebral hemorrhage; NLICH, nonlobar intracerebral hemorrhage; SNP, single‐nucleotide polymorphism; SVS, small vessel stroke.

### 3.2 No Causal effect of AAM on the risk of IS and its subtypes was revealed

**FIGURE 4:**
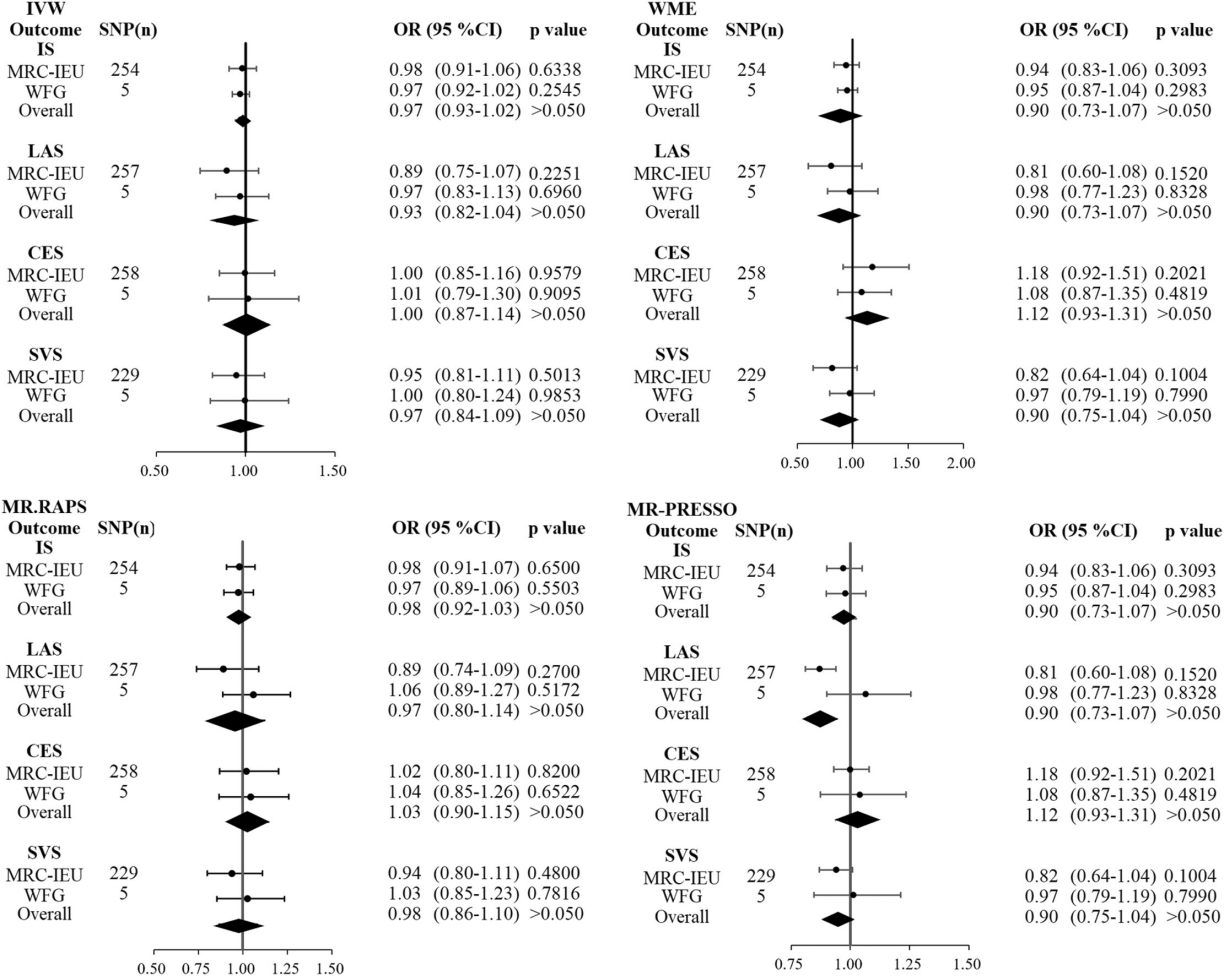
Causal relationships between AAM and IS with its subtypes by IVW, WME, MR.RAPS, MR‐PRESSO methods in the initial phase, replication phase, and meta‐analysis. Meta‐analysis of causality in the initial and replication phases did not confirm a causal relationship between AAM and IS. AAM: age at menarche. ICH: intracerebral hemorrhage, IVW, inverse‐variance‐weighted; NLICH, nonlobar intracerebral hemorrhage; SNP(*n*), SNP number; WFG, within‐family GWAS; WME, weighted median estimation.

The initial phase involved using a random effect model to examine whether AAM affected the IS and its subtypes in light of the presence of heterogeneity (Table 3). For IS (Figure 4), no causal relationship between AAM and risk of IS was found using the IVW method (OR = 0.98, 95% CI: 0.91–1.06, *p* = 0.64), MR‐PRESSO (OR = 0.97, 95% CI: 0.90–1.05, *p* = 0.48), MR.RAPS (OR = 0.98, 95% CI: 0.91–1.07, *p* = 0.65), WME (OR = 0.94, 95% CI: 0.83–1.06, *p* = 0.31), and MR‐Egger regression (OR = 0.84, 95% CI: 0.68–1.05, *p* = 0.12), respectively. Additionally, using the IVW technique, no causal link was shown for the subtypes of IS for LAS (OR = 0.89, 95% CI: 0.75–1.07, *p* = 0.89), CES (OR = 1.00, 95% CI: 0.85–1.16, *p* = 0.96), and SVS (OR = 0.95, 95% CI: 0.81–1.11, *p* = 0.50). However, weak evidence was found using the MR‐Egger regression technique between AAM and a decreased incidence of SVS (OR = 0.59, 95% CI: 0.38–0.92, *p* = 0.02).

Additionally, no causal relationships between AAM and the risk of IS (OR = 0.97, 95% CI: 0.83–1.13, *p* = 0.70) and its subtypes LAS (OR = 0.97, 95% CI: 0.92–1.02, *p* = 0.26), CES (OR = 1.02, 95% CI: 0.79–1.30, *p* = 0.91), and SVS (OR = 1.00, 95% CI: 0.80–1.24, *p* = 0.99) by IVW method were discovered using data from the within‐family GWAS consortium in the replication phase. Furthermore, a causal link between AAM and IS and its subtypes was not established in the meta‐analysis of data from the MRC‐IEU collaboration and within‐family GWAS consortium (Table 3 and Figure 4).

### 3.3 The results of the sensitivity analysis further attest to the reliability of these findings

As can be shown in Table 4, there was no evidence of heterogeneity (*p* > 0.05) or pleiotropy (*p* > 0.05) between AAM and the risks of ICH, NLICH, or LICH in the initial and replication phases. The replication phase data did not reveal any heterogeneity or pleiotropy between AAM and the risks of IS, LAS, CES, or SVS (*p* > 0.05). A random effects model was employed to lessen the influence of heterogeneity as suggested by the results because the causal association between AAM and IS and its subtypes in the initial phase was found to be heterogeneous (*p* < 0.05). Additionally, only the SVS showed heterogeneity in the MR‐Egger regression, and no outliers were observed although the *p*‐value for the MR‐PRESSO global test was <0.05.

**TABLE 4:** Outcome of sensitivity analysis for AAM and risk of ICH and IS in two‐sample Mendelian randomization study.

	Heterogeneity test		Pleiotropy test		Global test		*I* ^2^ (GX)%
	*Q*‐statistic	*p*	Egger intercept	*p*	RSSobs	*p*	
MRC‐IEU							
ICH	167.8	0.170	−0.0058	0.680	183.4	0.370	99
NLICH	162.5	0.300	−0.0035	0.830	182.3	0.405	99
LICH	140.5	0.740	−0.0230	0.190	158.0	0.860	99
IS	391.6	0.001	0.0030	0.140	332.6	0.004	99
LAS	307.5	0.010	−0.0035	0.480	331.9	0.001	99
CES	318.3	0.004	−0.0080	0.060	330.2	0.004	99
SVS	253.5	0.040	0.0093	0.026	265.6	0.033	99
WFG							
ICH	0.140	0.997	0.0107	0.935	1.31	0.982	99
NLICH	0.138	0.998	0.0323	0.837	5.537	0.759	99
LICH	0.880	0.927	−0.0079	0.963	1.507	0.980	99
IS	1.552	0.817	0.0208	0.379	13.325	0.179	43
LAS	2.565	0.633	−0.0410	0.460	9.080	0.430	90
CES	9.363	0.053	0.0007	0.993	13.239	0.208	86
SVS	7.622	0.107	0.0908	0.110	16.196	0.111	85

*Note*: No significant heterogeneity or pleiotropy in either the initial or replication phases influenced the causal relationship between AAM and risk of ICH, NLICH.

Abbreviations: AAM, age at menarche; CES, cardioembolic stroke; ICH, intracerebral hemorrhage; IS, ischemic stroke; LAS, large artery stroke; NLICH, nonlobar intracerebral hemorrhage; SVS, small vessel stroke.

## 4 DISCUSSION

To our knowledge, this is the first MR study to explore the potential causal relationship between AAM and ICH/IS and its subtypes. A two‐stage MR analysis of a sizable sample size of GWAS was conducted to establish a more trustworthy causal link between AAM and ICH, IS, and their subtypes. We then used meta‐analysis to combine the causal effects of the two stages. Our MR investigation offers proof that AAM has a direct causal relationship with the chance of developing ICH and its subtype NLICH. However, we were unable to identify a causal connection between AAM, LICH, IS, or the majority of its subtypes. These serve as a reference for comprehending the influence and probable processes of hereditary variables connected to AAM on cerebrovascular illness.

There are a growing number of epidemiological studies examining how gender affects the incidence, prevalence, and subtypes of IS and ICH.^13^, ^14^, ^15^, ^29^ Gender differences in ICH were investigated in 2017 in a prospective study with 2212 patients, and results revealed that women were more likely predisposed with LICH than males.^29^ Our conclusion that AAM may be causally linked to a lower risk of NLICH may be supported by this finding. Previous research has shown that the effects of AAM on metabolic disease and cardiovascular disease are very closely tied to the impact of estrogen.^14^, ^15^, ^30^, ^31^ Early AAM may cause early initiation of estrogen exposure, elevated blood levels of estradiol, and potentially compromised vascular elasticity and coagulation.^30^, ^32^ In some cases, extending the time that people are exposed to estrogen may lower their risk of developing endometrial cancer. The body, however, is exposed to estrogen too early if the AAM is premature, leading to a significant estrogen accumulation impact.^31^ This is comparable to the effects of oral contraceptives^33^ or synthetic estrogen replacement therapy,^34^, ^35^ which lengthen exposure time and dose, increasing vascular system flexibility, impairing coagulation, and raising the risk of ICH. It has not been advantageous for AAM to gradually increase among women as a result of social and economic improvement. In addition to AAM affecting estrogen, factors such as the timing of menopause and the duration of the entire menstrual period may influence the causal relationship between AAM and ICH by influencing factors such as the duration of exposure to estrogen and progesterone. Generally speaking, this could raise the likelihood of ICH in the female population. On the contrary, late AAM may improve the long‐term health of women by reducing the risk of ICH in women through decreasing the effects of estrogen and progesterone.

AMM may influence obesity and hypertension, in addition to the direct effects previously indicated, to affect the risk of ICH, particularly NLICH. According to one study, late AAM may somewhat lower the risk of hypertension. This observational study showed that 1 year of late AAM would reduce the incidence of hypertension by roughly 3%.^36^ They have looked into the basic mechanisms that connect them. Adolescent obesity is frequently present in early AAM patients.^37^ Adipose tissue buildup boosts the activity of the enzyme aromatase, which transforms testosterone into estrogen and causes an increase in estrogen levels in the female population.^31^ The buildup of estrogen will make hypertension more likely and consequently leading to high risk of ICH.^38^ AAM may also prematurely trigger NLICH through heightened sympathetic nervous system activity, the renin‐angiotensin‐aldosterone system, increased peripheral vascular resistance, or gonadotropin effects on blood pressure.^4^ The most frequent risk factor for ICH is hypertension, which frequently causes ICH in the putamen, basal ganglia, and other areas due to rapid changes in blood pressure and the area where the bleeding is occurring is an NLICH area. In light of this, the low risk of late AAM‐induced hypertension may contribute in some ways to the low risk of ICH and particularly NLICH.

Additionally, early AAM is linked to high BMI in adults, which has a variety of effects on the body in adults.^39^ High prepubertal BMI in females results in significantly higher plasma total estradiol levels, which would also have an estrogenic compounding effect and raise the risk of ICH in adulthood.^40^ Furthermore, earlier observational studies support our findings.^9^ Obesity has been linked to insulin resistance, renin‐angiotensin system activation, increased adipocyte‐derived resistance, and increased leptin effects, but not LICH, which has been suggested to increase the risk of profound ICH but not LICH.^41^, ^42^, ^43^ These are all strong arguments in favor of drawing a connection between AAM and ICH, NLICH.

In addition, we found no causal relationship between AAM and IS and its subtypes, unlike previous observational studies. This may be due to the different aims and methods of analysis, as this study was conducted from a genetic perspective to reveal the causal relationship between AAM and IS, ICH, and their subtypes. In addition, this may suggest that previous observational studies between AAM and IS may have been influenced by confounding factors and other factors that interfered with the direct determination of causality.

In the initial phase of investigating the causal relationships between AAM and IS and their subtypes, there were potential effects of heterogeneity and pleiotropy. However, we used a random effects model to mitigate the impact of heterogeneity on causality. The MR‐PRESSO outliers test was used to screen for outliers up until no outliers were discovered, and exclusions were made to lessen the impact of the existence of outliers on causality. As a result, outliers have a much smaller effect on causality. Additionally, the reliability of the causality in the initial phase is further supported by the fact that the causality in our replication phase was the same as in the initial phase. Second, information for AAM was extracted from the open GWAS database. For further mechanistic analysis, we were unable to locate data on this population's nutritional status, BMI, waist circumference, height, and other variables. The association between AAM and ICH in other ethnic groups needs to be further validated as there are some regional differences in morbidity and mortality between IS and ICH. Finally, all GWAS data included in this study were of European origin.^44^, ^45^ As a result, we ought to exercise caution when extrapolating the findings to other ethnic groups.

## 5 CONCLUSION

In conclusion, our research offers genetic support for a causal link between AAM and ICH, especially NLICH in European population. This finding offers some guidance for preventing ICH risk, particularly NLICH risk in the female population, but further experimental and clinical research is required to establish the underlying mechanisms.

## AUTHOR CONTRIBUTIONS

The research was planned, and the manuscript's organization was chosen by Le Zhang and Xuelun Zou. References were chosen, and writing assistance was provided by Xuelun Zou, Leiyun Wang, and Sai Wang. Data from GWAS were gathered by Leiyun Wang. These Mendelian randomization study's findings were examined with assistance from Xuelun Zou and Leiyun Wang. The article was revised and polished with help from Le Zhang. The article's submission and all of its writers' contributions were both accepted.

## CONFLICT OF INTEREST STATEMENT

The authors do not declare any potential conflict of interest relevant to this article.

## Supporting information


Table S1‐S2.

Table S1.

Table S2.

## Data Availability

The data that support the findings of this study are openly available in IEU‐GWAS at https://gwas.mrcieu.ac.uk/datasets/?trait__icontains=Body%20mass%20index.
