# Supplementary material for: Mendelian randomization study and meta‐analysis exploring the causality of age at menarche and the risk of intracerebral hemorrhage and ischemic stroke
Source: CNS Neurosci Ther. 2023 May 11;29(10):3043–52. doi: 10.1111/cns.14245 (PMC10493675; doi:10.1111/cns.14245)
Supplement: Supplementary file 1 — Table S1‐S2. [file CNS-29-3043-s002.docx]

Supplementary Table 1 is “The Basic Information about all selected SNPs. SNPs: Single nucleotide polymorphisms, ICH: intracerebral hemorrhage, IS: ischemic stroke, SVS: small vessel stroke, CES: cardioembolic stroke, LAS: large artery atherosclerosis stroke”.

Supplementary Table 2 is entitled “Information on the excluded instrumental variables for this study, ICH: intracerebral hemorrhage, LICH: lobar intracerebral hemorrhage, NLICH: non-lobar intracerebral hemorrhage, IS: ischemic stroke, SVS: small vessel stroke, CES: cardioembolic stroke, LAS: large artery atherosclerosis stroke”.
